# Provisional Dynamic Hinged External Fixation Enables Arthroscopy‐Assisted Circular Frame Reconstruction of Complex Tibial Plateau Fractures

**DOI:** 10.1002/ars2.70010

**Published:** 2026-04-30

**Authors:** Ilan Y. Mitchnik, Uri Hazan, Mohammad Shalabi, Yaron Golzman, Najib Chacar, Yakov Odessky, Vitali Aranbitski, Yiftah Beer

**Affiliations:** ^1^ Department of Orthopaedic Surgery Shamir Medical Center Beer Yaakov Israel; ^2^ Gray Faculty of Medical & Health Sciences Tel Aviv University Tel Aviv Israel

## Abstract

**Purpose:**

To describe the surgical technique and early operative outcomes of using a provisional dynamic external fixator to facilitate arthroscopic‐assisted reduction of complex tibial plateau fractures.

**Methods:**

Between 2014 and 2024, we conducted a retrospective single‐center case series of Schatzker IV‐VI tibial plateau fractures treated with provisional dynamic external fixation and arthroscopy‐assisted circular frame fixation, with a minimum 1‐year follow‐up. The dynamic fixator permitted knee flexion during definitive surgery, facilitating arthroscopic reduction. Collected variables included perioperative metrics, hospitalization course, 1‐year radiographic outcomes, complications, time to union, and knee range of motion.

**Results:**

Eleven patients met criteria and were predominantly healthy middle‐aged (mean 46.27 years) men (78%). Continuous passive motion began immediately after provisional fixation. Median operative time was 1 hour and 17 minutes for the dynamic frame and 3 hours and 4 minutes for the arthroscopy‐assisted definitive procedure, with low estimated blood loss at both stages (median 5 to 25 mL). Definitive fixation occurred a median of 5 days after injury; median hospital stay was 12 days; 83% were discharged home. At 1 year, mean articular depression was 2.25 mm, condylar widening 0.25 mm, and angulation of 4.46°; the median Rasmussen score was 12. Median time to union was 13 weeks and 3 months for full weight‐bearing. After 1 year, range of motion was 0° to 115° on average. Complications within 1 year, included infection in 5/11 (45%)—4 pin‐site infections treated with oral antibiotics, and 1 deep infection requiring debridement, foot drop 1/11 (9%) and chronic pain 1/11 (9%); no revision surgeries occurred.

**Conclusions:**

This case series shows that provisional fixation with a dynamic hinged external fixator for complex tibial plateau fractures permitted knee motion during the interval before definitive surgery and preserved access for arthroscopy‐assisted reduction without requiring frame removal. At 1 year, radiographic outcomes were good, knee range of motion was near full, and there was a low rate of serious complications.

**Level of Evidence:**

Level IV, retrospective therapeutic case series.

## Graphical abstract

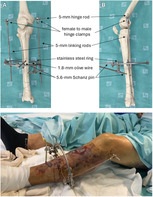

Tibial plateau fractures account for up to 2% of adult long bone fractures.^1^ They occur predominantly in young men following high‐energy injury mechanisms such as motor‐vehicle accidents or falls from height.^2^ Operative management of these fractures is complicated by the thin soft‐tissue envelope surrounding the proximal tibia, as its dissection risks wound dehiscence and infection.^3^ Treatment goals are accurate articular fracture reduction, normal limb alignment, and sufficient stability to permit early knee range of motion (ROM).^3^

Open reduction and internal fixation (ORIF) with plate osteosynthesis is the most common strategy to achieve these treatment goals.^3^ However, plate thickness and soft‐tissue stripping may exacerbate soft‐tissue damage further.^3^ Thus, some medical centers employ a staged management protocol in which a temporary provisional external fixator maintains alignment and stability until swelling resolves, followed by definitive fixation.^4^, ^5^ Provisional external fixation of tibial plateau fractures can be performed with trans‐knee monolateral fixators or with circular fine‐wire frames (Ilizarov constructs).^3^ When used definitively, circular frames offer comparable outcomes to plate fixation while permitting earlier weight‐bearing and less soft‐tissue complications.^6^, ^7^, ^8^, ^9^, ^10^, ^11^

In either method of definitive fixation, accurate intra‐articular reduction is challenging.^3^ Arthroscopy, in contrast with fluoroscopy or an arthrotomy, provides direct visualization of the joint surface and has been associated with smaller residual gaps and faster rehabilitation.^12^ However, the bulky rings of circular frames obstruct arthroscopic portal and instrument maneuvers. To address this, we designed a dynamic provisional external fixation construct that couples a partial circular frame on the tibia with a femoral trans‐condylar hinge (Figure 1). This technique was developed at our institution for staged management for tibial plateau fractures. The construct applies ligamentotaxis to maintain alignment, permits controlled knee motion during the soft‐tissue recovery interval, and is easily converted to a full circular frame at the definitive stage while allowing unobstructed arthroscopic reduction. The purpose of this study was to describe the surgical technique and early the operative outcomes of using a provisional dynamic external fixator to facilitate arthroscopic‐assisted reduction of complex tibial plateau fractures. We hypothesized that, in complex tibial plateau fractures, a dynamic hinged provisional external fixator would safely maintain alignment while permitting immediate knee motion and preserving arthroscopic access, thereby facilitating accurate reduction at definitive circular frame fixation with acceptable 1‐year radiographic outcomes.

**FIGURE 1:**
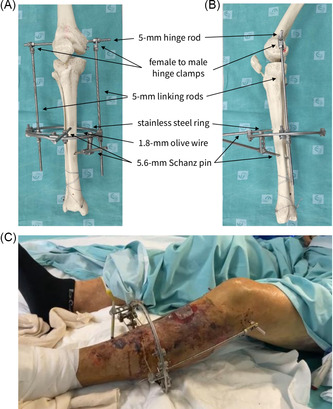
Provisional dynamic external fixation components. (A and B) Photographs of a right knee synthetic bone model demonstrating the provisional dynamic external fixator design. A stainless‐steel circular tibial ring is connected to the femur via a 5‐mm trans‐condylar hinge rod fixed with female‐to‐male hinge clamps. The ring is anchored to the tibia with two 5.6‐mm Schanz pins and 2 tensioned 1.8‐mm olive wires. The hinged construct permits sagittal‐plane knee motion and maintains coronal‐plane alignment through ligamentotaxis. (C) Bedside clinical photograph of a supine patient with a left tibial plateau fracture and soft‐tissue injury treated using the dynamic fixator. The hinge permits knee flexion during the soft‐tissue recovery interval, allowing early motion and preserving arthroscopic access at the time of definitive fixation.

## METHODS

### Study Design and Setting

This is a retrospective consecutive case series of patients with complex tibial plateau fractures managed by a staged protocol comprising provisional dynamic external fixation followed by arthroscopy‐assisted circular frame reconstruction between 2014 and 2024. All procedures were performed at a single tertiary hospital during the study period. Eligible cases were retrieved from an institutional database using *International Classification of Diseases, Ninth Revision* codes 823.0 and 823.1 (fracture of upper end of tibia and fibula closed/open). Two orthopaedic trauma surgeons (U.H., V.A.) reviewed index radiographs and assigned appropriate Schatzker classifications (I, II, III, IV, V, or VI).^13^ Inclusion criteria encompassed age ≥18 years, complex fracture patterns as classified by Schatzker class IV, V, or VI, procedures where provisional dynamic external fixation was used, and minimum 1 year of follow‐up. We then excluded patients whose secondary definitive circular frame fixation did not involve arthroscopic assistance (usually due to unavailability of a surgeon proficient in arthroscopies). The study was approved by an institutional review board, with the requirement for informed consent waived due to its retrospective nature (approval no. 0125‐24‐ASF).

### Indications for Surgery

Emergent external fixation was indicated for patients with complex tibial plateau fractures (Schatzker IV, V, and VI) with significant displacement, gross malalignment, or open fractures.

### Staged Management Protocol

Patients with surgical indications were taken emergently to the operating room for provisional external fixation. Whenever an on‐call surgeon experienced in circular frame constructs (Y.O., V.A., U.H.) was available, a dynamic external fixator was applied. Only patients who received a dynamic external fixator by a trained provider were included in this study; cases treated with a trans‐knee monolateral frame were excluded from this study. After provisional fixation, patients remained hospitalized for soft‐tissue surveillance and definitive fixation planning. Patients treated with the dynamic external fixator began continuous passive motion therapy (0° to 30° arc) on postoperative day 1 to maintain knee ROM. Definitive surgery with arthroscopy‐assisted reduction and complete circular frame fixation was scheduled ideally within a week.

### Provisional Dynamic External Fixation: Surgical Technique

**FIGURE 2:**
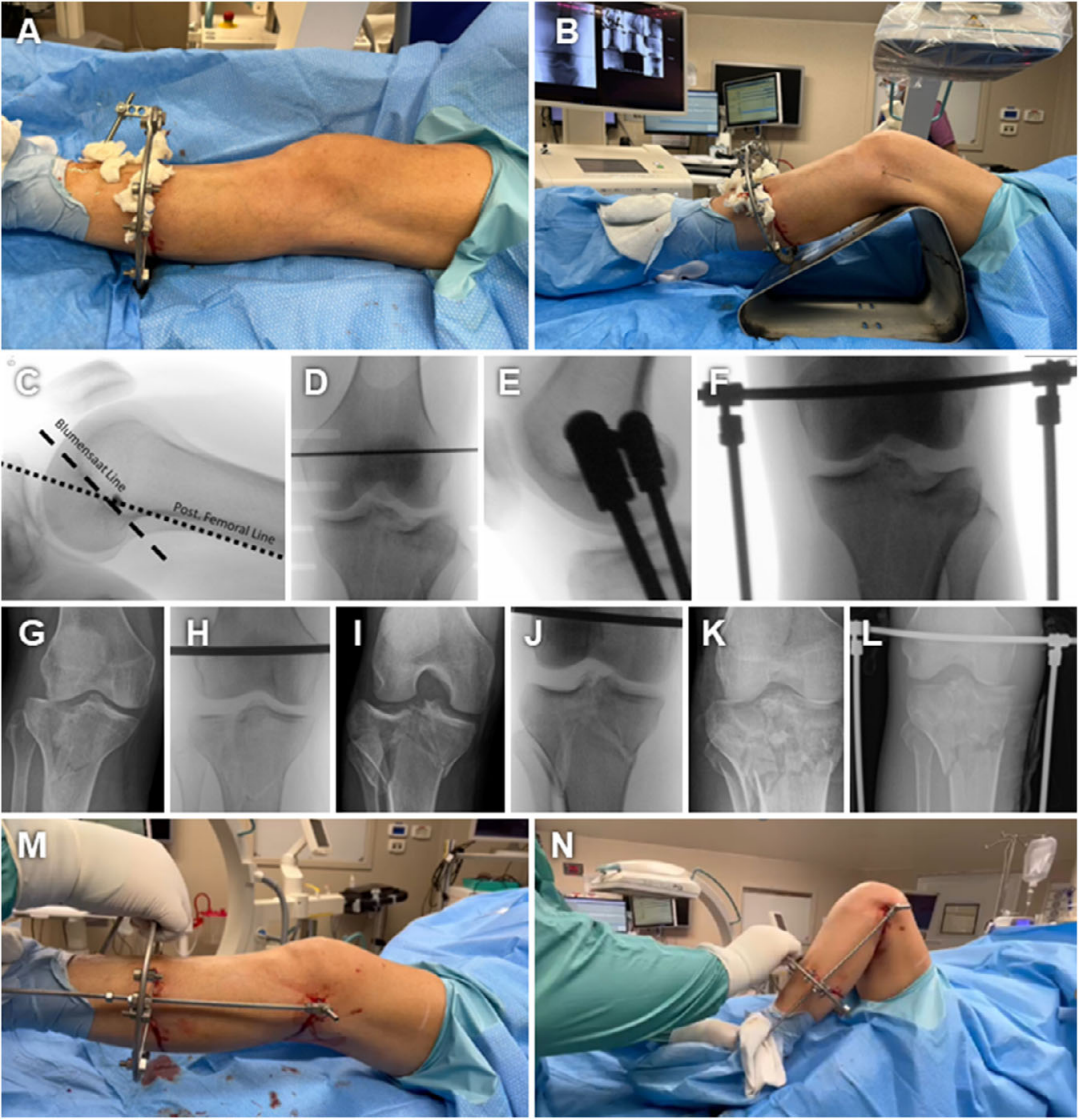
Construction of the provisional dynamic external fixator. This intraoperative sequence demonstrates the assembly and function of a motion‐preserving dynamic external fixator in a 40‐year‐old woman with a left tibial plateau fracture. (A) Clinical photograph showing a 160‐mm circular tibial ring fixator mounted on the middle third of the left tibia using Schanz pins and tensioned wires, with the patient supine. (B) Guidewire insertion through the distal femur from medial to lateral under fluoroscopic guidance with the leg supported in flexion over a triangular bolster. (C) Lateral fluoroscopic view identifying the knee's center of rotation at Schöttle's point, just proximal and anterior to the intersection of the Blumensaat line (thicker dashed line) and posterior femoral cortex (thinner dashed line). (D) Anteroposterior fluoroscopic view showing bicortical placement of the 1.8‐mm guidewire; this was then followed by reaming with a 4.5‐mm drill to create a trans‐condylar tunnel through which a hinge rod was passed. (E) Lateral fluoroscopic image of the hinge rod connected to the tibial ring with female‐to‐male hinges. (F) Anteroposterior fluoroscopic view after traction is applied across the hinge, distracting the joint line to aid ligamentotaxis. (G and H) Anteroposterior fluoroscopic images in a 51‐year‐old woman with a Schatzker IV fracture: before traction (G) and after traction (H). (I and J) Same views in a 27‐year‐old man with a Schatzker VI fracture: before (I) and after traction (J). (K and L) Same views in a 26‐year‐old man with a Schatzker VI fracture: before (K) and after traction (L). (M and N) Clinical photographs of the final fixator configuration in the index patient (A‐F), showing knee in full extension (M) and flexion (N), demonstrating the functional arc allowed during provisional fixation.

After induction of anesthesia, we administered prophylactic antibiotics and positioned the patient supine on a radiolucent fracture table. A tourniquet was not used. The limb was prepped and draped in sterile fashion. Open wounds, when present, underwent irrigation and debridement before dynamic external fixator assembly. Next, the following stepwise procedure was performed:

Step 1 ‐ Tibial ring (Figure 2A).

A single 160‐ or 180‐mm stainless‐steel ring was mounted on the middle tibia using one 1.8‐mm olive wire tensioned at 120‐kg force, one 1.8‐mm Kirschner wire, and two 5.6‐mm hydroxyapatite‐coated Schanz pins, oriented 60° to each other. Pin and wire placement respected the peroneal and tibial neurovascular safe zones.

Step 2 ‐ Femoral trans‐condylar hinge (Figure 2B‐D).

The patient's leg was placed on a triangular bolster (Figure 2B), and under fluoroscopic guidance, Schöettle's point was identified just proximal and anterior to the intersection of the posterior femoral cortex and Blumensaat line (Figure 2C). This point is referenced as the knee's center of rotation for the dynamic external fixator. First, a 1.8‐mm guidewire was advanced bicortically and over‐reamed with a 4.5‐mm cannulated drill (Figure 2B and D). Next, a 5‐mm stainless‐steel connecting rod (standard circular frame link) was inserted to function as a trans‐condylar hinge (Figure 2E and F). Correct trajectory was confirmed on anteroposterior and lateral images.

Step 3 ‐ Frame assembly and distraction (Figure 2E‐L).

The femoral hinge rod was linked to the tibial ring with female‐to‐male hinge clamps (Figure 2E and F). Gradual distraction (2 to 3 mm) restored length and alignment (Figure 2F‐L) while preserving 0° to 130° controlled knee flexion (Figure 2M and N). Final fluoroscopy confirmed coronal and sagittal alignment. Pin sites were dressed with povidone‐iodine‐soaked gauze and covered by a bandage.

### Definitive Circular Frame Fixation With Arthroscopy‐Assisted Reduction: Surgical Technique

**FIGURE 3:**
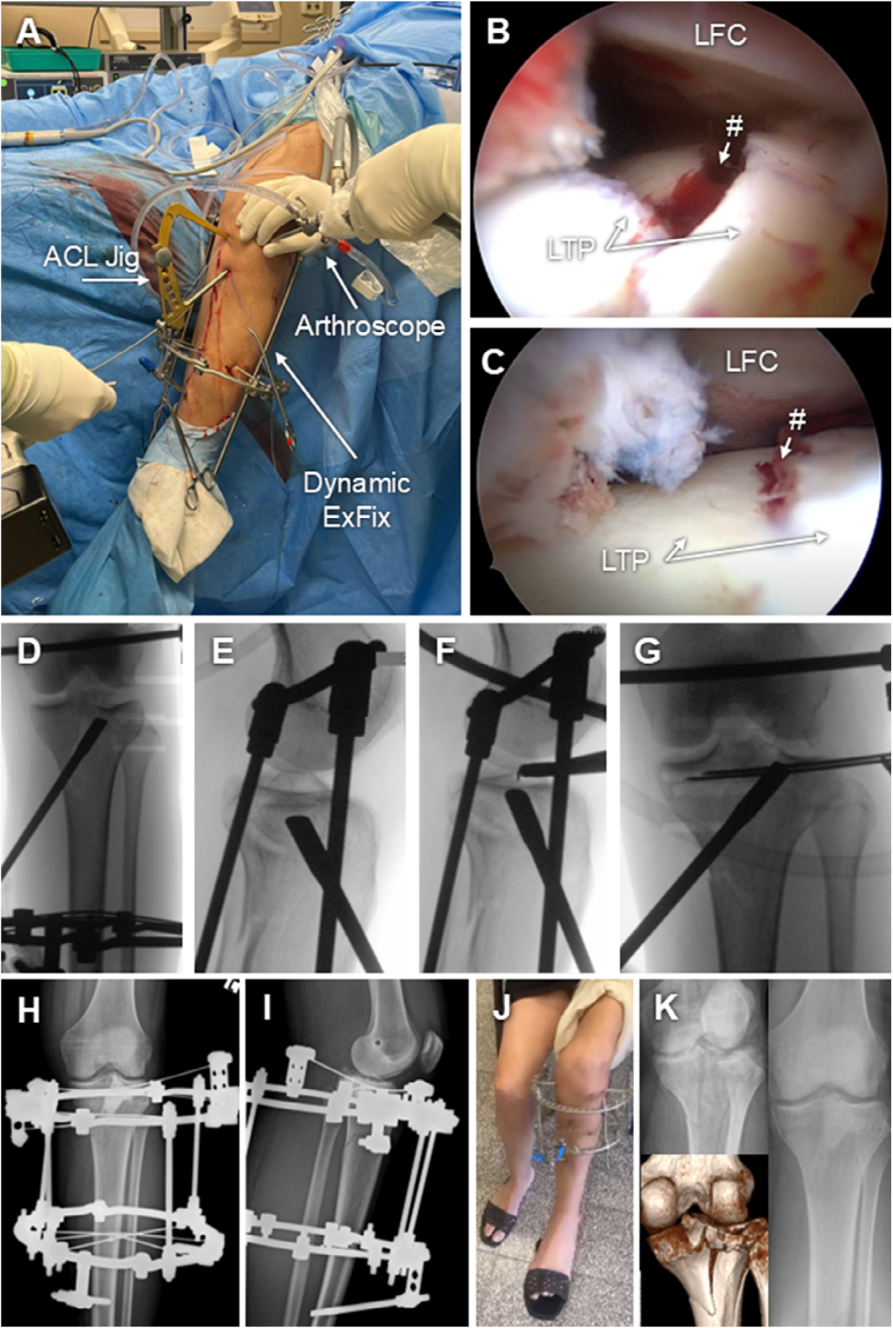
Arthroscopy‐assisted reduction of tibial plateau fracture facilitated by a dynamic external fixator. This sequence shows the same patient depicted in Figure 2A‐K, now undergoing definitive circular frame fixation with arthroscopy‐assisted reduction while still in the provisional dynamic external fixator. (A) Intraoperative image of the left knee during arthroscopy through the anterolateral portal. The dynamic fixator (Dynamic ExFix) permits knee flexion and unobstructed portal access without frame removal. An anterior cruciate ligament targeting jig (ACL Jig) is introduced to guide fracture reduction. (B) Arthroscopic view (anterolateral portal) showing a depressed lateral tibial plateau fracture line with an articular gap. (#, fracture line; LTP, lateral tibial plateau; LFC, lateral femoral condyle). (C) Same view after disimpaction and reduction of the fragment. (D) Anteroposterior fluoroscopic image of a bone tamp being inserted through the tibial tunnel created with the ACL jig. (E) Lateral fluoroscopic view showing the tamp positioned beneath the depressed lateral plateau. (F) Same view after elevation of the fragment using the tamp. Arthroscopic confirmation of the reduction is seen (scope in joint). (G) Anteroposterior fluoroscopic view showing the reduced fragment secured with 2 Kirschner wires. (H) Final anteroposterior radiograph demonstrating circular frame construct and metaphyseal void filled with tricalcium phosphate. (I) Lateral radiograph of the same final construct. (J) Photo of the final construct. (K) Top left: preoperative anteroposterior radiograph showing the index injury: a bicondylar tibial plateau fracture (Schatzker V); Bottom left: a 3D reconstruction computed tomography image of the fracture showing the depressed lateral fragment (seen from posterior view); Right: the final anteroposterior radiograph at 1‐year follow‐up.

We positioned the patient supine on a radiolucent fracture table, prepped and draped the extremity with chlorhexidine, and administered prophylactic antibiotics and tranexamic acid. No tourniquet was used. Through standard anterolateral and anteromedial portals, we performed diagnostic arthroscopy (Figure 3A), evacuated the hemarthrosis, and debrided as needed to expose the fracture zones (Figure 3B). Meniscal pathology was treated with repair or meniscectomy as indicated.

Articular depression was addressed using a targeted reduction technique under combined arthroscopic and fluoroscopic guidance. Through a small incision at the pes anserinus, we introduced an anterior cruciate ligament reconstruction jig to guide a tibial tunnel toward the depressed fragment (Figure 3A). A guidewire was passed through the jig sleeve, then overdrilled to create a working tunnel. A bone tamp was advanced through the tunnel to disimpact and elevate the articular surface under direct arthroscopic visualization (Figure 3C‐E). The fragment was provisionally fixed with Kirschner wires, which were later incorporated into the definitive frame. The resulting metaphyseal void was filled with tricalcium phosphate to support the subchondral bone (Figure 3H). When present, condylar widening was corrected using a percutaneous bone‐reduction clamp.

A proximal tibial circular frame was assembled to maintain the correction (typically a 200‐mm ring), with olive wires holding fragment reductions and buttressing the reduced surface (Figure 3H and I). Additional Kirschner wires were used for ring fixation if necessary. Reduction accuracy was confirmed by a deliberate “double‐check”: direct arthroscopic inspection together with fluoroscopy, before finalizing fixation (Figure 3C).

We then released distraction between the femoral trans‐condylar rod and tibial ring and removed the femoral hinge. Arthroscopy portals were closed with absorbable sutures. Pin sites were dressed with alcohol‐soaked gauze, and an elastic/Esmarch bandage was applied to maintain the foot in dorsiflexion.

### Data Collection, Variables, and Measures

Data were extracted from the medical center's electronic medical record system and picture archiving and communication system. Demographics included age, sex, and body mass index. Comorbidity burden was calculated using the Charlson Comorbidity Index.^14^ Injury data included mechanism, open or closed fracture descriptions, and Schatzker classification.^13^ Injury Severity Scores were derived from documented associated injuries.^15^ Procedure variables included operative time (hh:mm) and estimated blood loss (mL) for both the provisional dynamic external fixator and the definitive circular frame procedure. Additional information about time to definitive surgery (days), length of hospital stay (days) and discharge locations (rehabilitation or home) were also collected. One‐year postoperative clinic follow‐up data included ROM measurements, fracture union time (weeks), time to weight‐bearing (months), and occurrence of postoperative complications such as surgical‐site infections (including pin‐site infections), foot drops (or peroneal nerve injuries), and chronic pain symptoms. In addition, records of repeat emergency department visits, hospital readmissions, and revision surgeries were reviewed. Radiographic assessments available at final 1‐year follow‐up used standard anteroposterior weight‐bearing knee radiographs. We used the criteria described by Rasmussen et al.^16^ and measured articular depression (mm), condylar widening (mm), and the medial proximal tibial angle as varus/valgus angulation (90 − medial proximal tibial angle). We followed the measurement techniques described by Durakbasa et al. (measured by I.Y.M.).^17^ Rasmussen's radiological outcome scores can be interpreted in the following way: 0‐5 points are considered poor, 6‐11 points are fair, 12‐17 points are good, and 18 points are excellent.^16^

### Statistical Analysis

A descriptive analysis was performed and continuous variables were presented as means with standard deviations/errors or medians with interquartile ranges based on histogram distributions. Categorical variables were described as counts (n) and percentages (%). All statistical analyses were performed using IBM SPSS Statistics for Windows, version 25 (IBM, Armonk, NY).

## RESULTS

**FIGURE 4:**
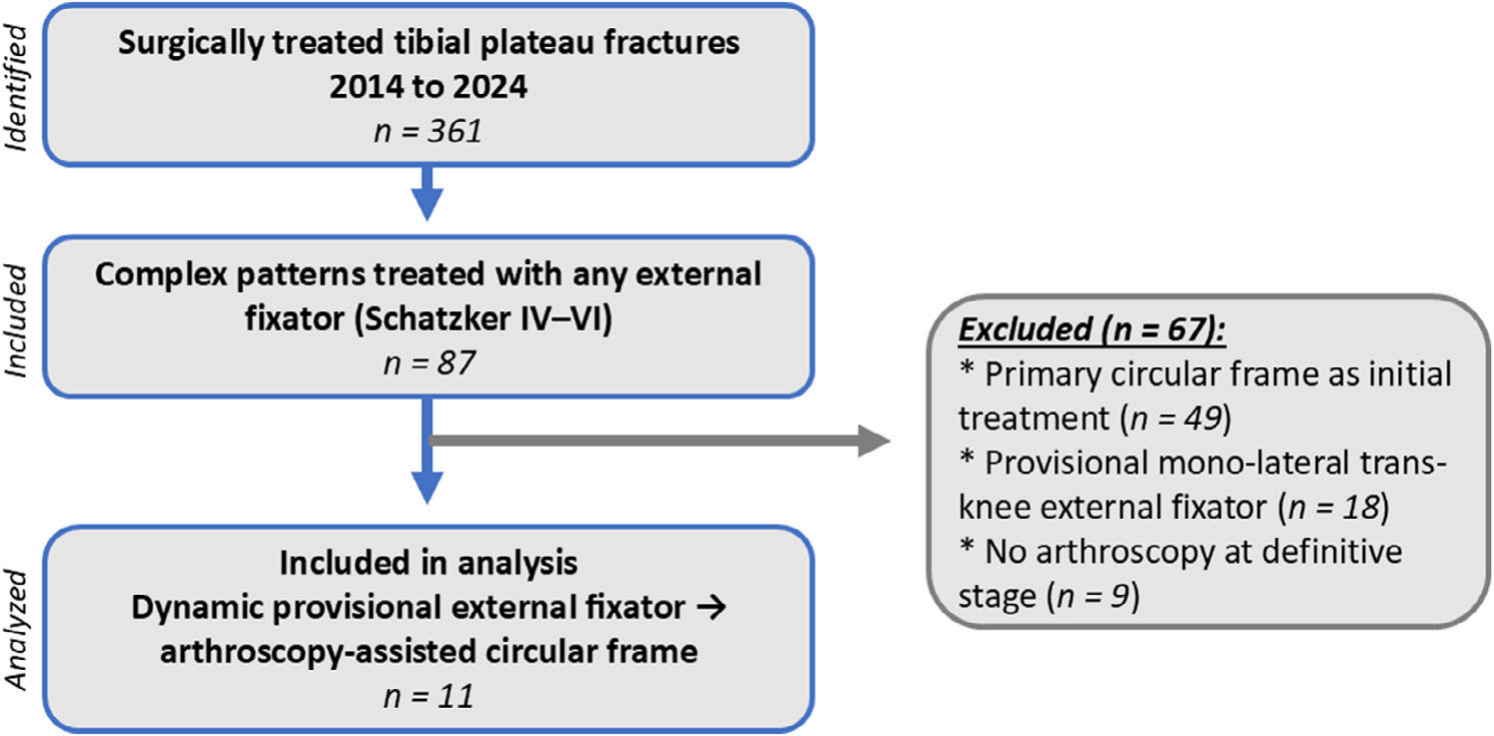
Flow diagram of cohort selection. This flow diagram outlines the selection process for the case series.

During the study time frame, we identified 361 cases of surgically treated tibial plateau fractures. Of these, 87 cases were complex fracture patterns (Schatzker IV, V, and VI) treated by external fixators of any kind. After excluding 49 cases treated with primary circular frame external fixators and 18 cases treated with provisional monolateral trans‐knee external fixators, 20 cases treated with provisional dynamic external fixation remained. Another 9 cases not managed with arthroscopic reductions at the definitive stage were further excluded from this case series. Overall, 11 cases of complex tibial plateau fractures treated with provisional dynamic external fixation and later with definitive circular frame external fixation with arthroscopy‐assisted reductions were included in this study (Figure 4).

Baseline characteristics are shown in Table 1. The cohort was predominately middle‐aged men with near‐normal body mass index and few comorbidities. Injuries were caused by either motor‐vehicle accidents or direct blows to the knee, with no open fractures recorded and low Injury Severity Score levels.

**TABLE 1:** Baseline Demographics and Injury Characteristics

Characteristics	Overall (n = 11)
Demographics	
Age, years, mean ± SD	46.27 ± 12.88
Female, n (%)	3 (27%)
Male, n (%)	8 (73%)
BMI, mean ± SD	25.78 ± 3.48
CCI, median (IQR)	1 (0 to 2)
Injury	
Mechanism, n (%)	
• Direct blow	6 (55%)
• Motor‐vehicle accident	5 (45%)
Open fracture, n (%)	0 (0%)
ISS, median (IQR)	4 (4 to 4)
Schatzker Class, n (%)	
• IV	3 (27%)
• V	2 (18%)
• VI	6 (55%)

BMI, body mass index; CCI, Charlson Comorbidity Index; ISS, injury severity score; IQR, interquartile range; SD, standard deviation.

Key operative and hospitalization metrics are summarized in Table 2. Median operative time was approximately between 1 hour and 1.5 hours for the provisional dynamic external fixation and almost 3 hours long for the arthroscopy‐assisted definitive circular frame fixation procedure. Both stages had low estimated blood losses. All patients were placed on continuous passive motion devices immediately after preliminary fixation as they awaited their definitive surgery. Definitive fixation occurred a median of 5 days (interquartile ranges = 4 to 7) after preliminary dynamic external fixation, hospital stay was a median of 12 days, and most patients were discharged home.

**TABLE 2:** Operative and Hospitalization Outcomes

Outcome	Overall (n = 11)
Preliminary dynamic external fixation	
Operative time, hh:mm, median (IQR)	01:17 (01:07 to 01:39)
Estimated blood loss, mL, median (IQR)	5 (0 to 20)
Definitive circular frame fixation with arthroscopy	
Operative time, hh:mm, median (IQR)	03:04 (02:51 to 03:26)
Estimated blood loss, mL, median (IQR)	25 (5 to 50)
Hospitalization	
Time to definitive fixation, days, median (IQR)	5 (4 to 7)
Length of stay, days, median (IQR)	12 (8 to 13)
Discharge destination, n (%)	
• Home	9 (82%)
• Rehabilitation	2 (18%)

IQR, interquartile range.

Table 3 presents radiographic measurements at 1 year. Mean articular depression was 2.25 mm, with minimal condylar widening of 0.25 mm and an angulation of 4.64°. Overall, the median Rasmussen radiographic score was 12. All patients achieved union, and union time was observed at a median of 13 weeks. Time to full weight‐bearing was recorded as a median of 3 months. After 1 year, ROM was observed at 0° to 115° on average (Table 3). Within 1 year, surgical‐site infections (including pin‐site infections) occurred in 5/11 (45%) of patients. Of these, 4/5 cases were minor pin‐site infections and managed with oral antibiotics while 1/5 was a deep infection that required wound debridement and irrigation. In addition, foot drop was identified in 1 patient, and 1 other patient developed a chronic pain syndrome. Emergency department visits were recorded in 6/11. These included visits 2/6 visits for postoperative pain, 3/6 visits for pin‐site infections, and 1/6 visit for deep wound infection. Readmissions were recorded in 4/11. These encompassed 3/4 readmissions for observation for suspected infections, and 1/4 was the patient requiring wound debridement and irrigation. Within 1 year, there were no revision surgeries.

**TABLE 3:** One‐Year Outcomes

Outcome	Overall (n = 11)
Radiographic measurements	
Articular depression, mm, mean ± SE	2.25 ± 0.57
Condylar widening, mm, mean ± SE	0.25 ± 0.84
Angulation, degrees, mean ± SE	4.64 ± 0.77
Rasmussen score, median (IQR)	12 (12 to 14)
Union time	
Weeks, median (IQR)	13 (13 to 14)
Time to weight‐bearing	
Months, median (IQR)	3 (3 to 3)
Range of motion (1 year)	
Degrees, mean ± SE	−0.18 ± 1.17 to 115.45 ± 3.12
Complications within 1 year, n (%)	
Pin‐site infections (managed by antibiotics)	4 (36%)
Deep infection (requiring debridement)	1 (9%)
Foot drop	1 (9%)
Chronic pain	1 (9%)
Emergency‐department visits	6 (55%)
Readmissions	4 (36%)
Revision surgery	0 (0%)

*Note*: Angulation reported as deviation from normal MPTA (medial proximal tibial angle), calculated as 90° − MPTA.

IQR, interquartile range; SE, standard error.

## DISCUSSION

The most important finding of this study is that a staged protocol using provisional dynamic external fixation for complex tibial plateau fractures was feasible. This approach permitted knee motion during the soft‐tissue recovery interval. It also allowed knee motion during definitive surgery with arthroscopy assistance. Such motion is not possible with traditional trans‐knee external fixators. This configuration preserves access for arthroscopy‐assisted reduction without requiring removal of the frame, thereby maintaining ligamentotaxis to facilitate reduction.

This study's staged management protocol utilizes arthroscopy as a “double‐check,” providing direct visualization to confirm the fluoroscopic reduction before the circular frame is locked. The advantages of arthroscopy‐assisted reduction are known to include more accurate restoration of the articular surface.^18^, ^19^ This is important because residual incongruity is associated with increased contact pressures and a higher risk of post‐traumatic osteoarthritis.^20^, ^21^ In addition, as reviewed by Burdin, open arthrotomies for complex tibial plateau fractures (Schatzker IV‐VI) are associated with soft‐tissue complications, thus supporting arthroscopy to assist in the articular surface reduction.^22^ Krause et al. described “fracturoscopy,” the insertion of an arthroscope through the open arthrotomy to aid articular reduction during tibial plateau ORIF.^23^ This was essentially an open‐assisted arthroscopy combined with plate fixation. Like Burdin, Krause et al. also note that ORIF entails soft‐tissue dissection, which may increase soft‐tissue complications. However, arthroscopy‐assisted reductions of tibial plateau fractures are especially challenging for complex bicolumnar fracture because rigid fixation is required to maintain fracture fragment positions. This issue is discussed by Chase et al. in their detailed surgical technique review for arthroscopy‐assisted tibial plateau fracture reductions.^18^ Our protocol avoids ORIF and instead uses a stable hinged external fixator to provide stability for arthroscopic reductions while minimizing additional soft‐tissue injury. Unlike a standard trans‐knee external fixator, this hinged device permits knee flexion, which can facilitate access to the posterior compartments while maintaining ligamentotaxis.

The motion‐preserving aspect of the dynamic external fixator we constructed is similar to hinged external fixators used after reconstructions of multiligamentous knee injuries.^24^ In studies by Stannard et al. and Fitzpatrick et al. of hinged/articulated external fixators for such injuries, the most emphasized benefit is the stability of these constructs.^25^, ^26^ This further supports the use of our device to provide stability during knee manipulation while performing arthroscopy. Additional benefits of hinged external fixators include prevention of arthrofibrosis due to prolonged knee immobilization, as described in a systematic review by Ayhan et al.^24^ At the 1‐year follow‐up, our patients showed a near complete ROM (full extension to approximately 115° of flexion, see Table 3). The early mobility that our device allows immediately after knee injuries may also benefit cartilage health because low‐frequency cyclic loading has been shown to slow osteoarthritis progression.^27^ This level of immediate knee mobility provided by the dynamic external fixator cannot be achieved with a conventional standard trans‐knee external fixation device. However, these chondroprotective effects appear to be less significant in post‐traumatic settings.^28^

Overall, this surgical technique resulted in a low rate of serious complications, and good radiographic outcomes. For tibial plateau fractures managed with circular frame fixations, the most common complication is infection. Pin‐site infection rates of up to 40% in circular frames for tibial plateau fractures have been previously reported.^29^ Our cohort had a similar rate, with 4/11 (36%) patients showing pin‐site infections. Deep infections have been reported to occur in up to 3.5% of these patients, and in our cohort, it occurred in a single patient.^30^ Peroneal injuries causing foot drops may occur in up to 6% of cases, while our cohort also had this complication in single patient.^31^ Chase et al. cautioned that fluid extravasation during arthroscopy for tibial plateau fractures can precipitate compartment syndrome, which we did not observe in our cohort.^18^ Acceptable radiographic outcomes include residual incongruities of 2 to 4 mm and up to 5° coronal malalignment.^32^ We measured radiographic outcomes in our cohort that were within these limits (Table 3). The overall Rasmussen radiological scores were comparable to those reported by Bormann et al. for complex tibial plateau fractures managed by ORIF.^33^

The implications of this surgical technique study are that medical centers practicing arthroscopy‐assisted reductions of tibial plateau fractures can adopt this dynamic external fixator to facilitate reductions in complex fracture patterns (Schatzker IV to VI). The device maintains alignment by ligamentotaxis and permits direct arthroscopic visualization of the articular surface, thereby double‐checking the fluoroscopic reduction. Furthermore, this device is a feasible alternative to a provisional static monolateral trans‐knee external fixation within a staged management protocol, while preserving knee motion as patients await definitive fixation. Finally, early passive motion of the knee may promote cartilage health and mitigate arthrofibrosis. We are planning a follow‐up comparative study to evaluate provisional dynamic external fixation against provisional static trans‐knee fixation to determine whether early joint motion confers measurable benefits.

### Limitations

This retrospective, single‐center case series included a small sample without a control cohort, limiting inference about the specific benefits of provisional dynamic external fixation. Sex‐based outcome comparisons were not performed due to insufficient sample size. We did not compare outcomes between cases treated with or without arthroscopy‐assisted reduction, as subgroup sizes were too small for meaningful statistical testing. Selection bias may have occurred, as use of the dynamic fixator depended on surgeon availability. Lastly, complications were identified through institutional records and may not reflect encounters at outside facilities.

## CONCLUSIONS

This case series shows that provisional fixation with a dynamic hinged external fixator for complex tibial plateau fractures permitted knee motion during the interval before definitive surgery and preserved access for arthroscopy‐assisted reduction without requiring frame removal. At 1 year, radiographic outcomes were good, knee ROM was near full, and there was a low rate of serious complications.

## DISCLOSURES

The authors (I.Y.M., U.H., M.S., Y.G., N.C., Y.O., V.A., Y.B.) declare that they have no known competing financial interests or personal relationships that could have appeared to influence the work reported in this paper.

## References

[1] Court‐Brown CM , Caesar B . Epidemiology of adult fractures: A review. Injury. 2006;37:691‐697. 16814787 10.1016/j.injury.2006.04.130

[2] Albuquerque RP , Hara R , Prado J , Schiavo L , Giordano V , Amaral NPD . Estudo epidemiológico das fraturas do planalto tibial em hospital de trauma nível I. Acta Ortop Bras. 2013;21:109‐115. 24453653 10.1590/S1413-78522013000200008 PMC3861961

[3] Tornetta P , III, Ricci WM , Ostrum RF , et al. Rockwood and Green's Fractures in Adults. 9th ed. Philadelphia, PA: Lippincott Williams & Wilkins; 2019.

[4] Egol KA , Tejwani NC , Capla EL , Wolinsky PL , Koval KJ . Staged management of high‐energy proximal tibia fractures (OTA Types 41). J Orthop Trauma. 2005;19:448‐455. 16056075 10.1097/01.bot.0000171881.11205.80

[5] Ryu SM , Yang HS , Shon OJ . Staged treatment of bicondylar tibial plateau fracture (Schatzker type V or VI) using temporary external fixator: Correlation between clinical and radiological outcomes. Knee Surg Relat Res. 2018;30:261‐268. 29554716 10.5792/ksrr.17.008 PMC6122938

[6] Babis GC , Evangelopoulos DS , Kontovazenitis P , Nikolopoulos K , Soucacos PN . High energy tibial plateau fractures treated with hybrid external fixation. J Orthop Surg Res. 2011;6:35. 21756337 10.1186/1749-799X-6-35 PMC3161896

[7] Ahearn N , Oppy A , Halliday R , et al. The outcome following fixation of bicondylar tibial plateau fractures. Bone Joint J. 2014;96‐B:956‐962. 10.1302/0301-620X.96B7.32837 24986951

[8] Canadian Orthopaedic Trauma Society . Open reduction and internal fixation compared with circular fixator application for bicondylar tibial plateau fractures. J Bone Joint Surg Am. 2006;88:2613‐2623. 17142411 10.2106/JBJS.E.01416

[9] Saad H , Adawy A , Meselhy M , Mostafa M . Treatment of tibial plateau fractures by circular external fixator. Benha Med J. 2021;38:996‐1008.

[10] Ahmed AS . Management of open complex tibial plateau fractures by Ilizarov fixator: Average follow‐up of 8.5 years. Egypt Orthop J. 2019;54:72.

[11] Ghimire A , Devkota P , Kumar Bhandari K , Kharel Y , Pradhan S . Ilizarov ring external fixation for complex tibial plateau fractures Fixação externa do anel de Ilizarov para fraturas complexas do platô tibial. Nepal Rev Bras Ortop. 2022;57:667‐674. 10.1055/s-0041-1739171 PMC9365483 35966423

[12] Ohdera T , Tokunaga M , Hiroshima S , Yoshimoto E , Tokunaga J , Kobayashi A . Arthroscopic management of tibial plateau fractures? Comparison with open reduction method. Arch Orthop Trauma Surg. 2003;123:489‐493. 12720016 10.1007/s00402-003-0510-3

[13] Schatzker J , McBroom R , Bruce D . The tibial plateau fracture. The Toronto experience 1968‐1975. Clin Orthop Relat Res. 1979;138:94‐104. 445923

[14] Charlson ME , Pompei P , Ales KL , MacKenzie CR . A new method of classifying prognostic comorbidity in longitudinal studies: Development and validation. J Chronic Dis. 1987;40:373‐383. 3558716 10.1016/0021-9681(87)90171-8

[15] Association for the Advancement of Automotive Medicine . Abbreviated injury scale: 2015 revision. 6th ed. Chicago, IL: Association for the Advancement of Automotive Medicine; 2018.

[16] Rasmussen PS . Tibial condylar fractures. Impairment of knee joint stability as an indication for surgical treatment. J Bone Joint Surg Am. 1973;55:1331‐1350. 4586086

[17] Durakbasa MO , Kose O , Ermis MN , Demirtas A , Gunday S , Islam C . Measurement of lateral plateau depression and lateral plateau widening in a Schatzker type II fracture can predict a lateral meniscal injury. Knee Surg Sports Traumatol Arthrosc. 2013;21:2141‐2146. 22956166 10.1007/s00167-012-2195-z

[18] Chase R , Usmani K , Shahi A , Graf K , Mashru R . Arthroscopic‐assisted reduction of tibial plateau fractures. Orthop Clin North Am. 2019;50:305‐314. 31084832 10.1016/j.ocl.2019.03.011

[19] Chen XZ , Liu CG , Chen Y , Wang LQ , Zhu QZ , Lin P . Arthroscopy‐assisted surgery for tibial plateau fractures. Arthroscopy. 2015;31:143‐153. 25125382 10.1016/j.arthro.2014.06.005

[20] Giannoudis PV , Tzioupis C , Papathanassopoulos A , Obakponovwe O , Roberts C . Articular step‐off and risk of post‐traumatic osteoarthritis. Evidence today. Injury. 2010;41:986‐995. 20728882 10.1016/j.injury.2010.08.003

[21] Oeckenpöhler S , Domnick C , Raschke MJ , Müller M , Wähnert D , Kösters C . A lateral fracture step‐off of 2mm increases intra‐articular pressure following tibial plateau fracture. Injury. 2022;53:1254‐1259. 35016775 10.1016/j.injury.2021.12.053

[22] Burdin G. Arthroscopic management of tibial plateau fractures: Surgical technique. Orthop Traumatol Surg Res. 2013;99:S208‐S218. 23347755 10.1016/j.otsr.2012.11.011

[23] Krause M , Preiss A , Meenen NM , Madert J , Frosch KH . “Fracturoscopy” is superior to fluoroscopy in the articular reconstruction of complex tibial plateau fractures—An arthroscopy assisted fracture reduction technique. J Orthop Trauma. 2016;30:437‐444. 26978133 10.1097/BOT.0000000000000569

[24] Ayhan EM , Levitt S , Abrams GD , Stannard JP , Medvecky MJ . The role of hinged external fixation in the treatment of knee dislocation, subluxation and fracture‐dislocation: A systematic review of indications. J Exp Orthop. 2025;12:e70275. 40390855 10.1002/jeo2.70275 PMC12086806

[25] Stannard JP , Sheils TM , McGwin G , Volgas DA , Alonso JE . Use of a hinged external knee fixator after surgery for knee dislocation. Arthroscopy. 2003;19:626‐631. 12861201 10.1016/s0749-8063(03)00125-7

[26] Fitzpatrick DC , Sommers MB , Kam BCC , Marsh JL , Bottlang M . Knee stability after articulated external fixation. Am J Sports Med. 2005;33:1735‐1741. 16093544 10.1177/0363546505275132

[27] Caravaggi P , Assirelli E , Ensini A , et al. Biomechanical‐based protocol for in vitro study of cartilage response to cyclic loading: A proof‐of‐concept in knee osteoarthritis. Front Bioeng Biotechnol. 2021;9:634327. 34012954 10.3389/fbioe.2021.634327 PMC8126668

[28] Eskelinen ASA , Florea C , Tanska P , et al. Cyclic loading regime considered beneficial does not protect injured and interleukin‐1‐inflamed cartilage from post‐traumatic osteoarthritis. J Biomech. 2022;141:111181. 35803036 10.1016/j.jbiomech.2022.111181

[29] Kumar J , Siddiqui AA , Katto MS , Jamil M , Wasim MA , Yaqoob U . Treatment of high‐energy intra‐articular fractures of tibia with Ilizarov external fixator in adults: A tertiary centre experience. Int J Clin Pract. 2021;75:e14488. 34115438 10.1111/ijcp.14488

[30] Madhvani KR , Fong A , Clark T , et al. Mid to long‐term outcomes of grade III‐B open tibial fractures definitively managed with a circular frame: A 13‐year prospective database study at a major trauma center. J Orthop Trauma. 2024;38:447‐451. 39007662 10.1097/BOT.0000000000002841

[31] Subramanyam KN , Tammanaiah M , Mundargi AV , Bhoskar RN , Reddy PS . Outcome of complex tibial plateau fractures with Ilizarov external fixation with or without minimal internal fixation. Chin J Traumatol. 2019;22:166‐171. 31072699 10.1016/j.cjtee.2018.11.003 PMC6543267

[32] Assink N , El Moumni M , Kraeima J , et al. Radiographic predictors of conversion to total knee arthroplasty after tibial plateau fracture surgery. J Bone Joint Surg Am. 2023;105:1237‐1245. 37196070 10.2106/JBJS.22.00500

[33] Bormann M , Bitschi D , Neidlein C , et al. Mismatch between clinical–functional and radiological outcome in tibial plateau fractures: A retrospective study. J Clin Med. 2023;12:5583. 37685650 10.3390/jcm12175583 PMC10488212

